# Prevalence and antimicrobial susceptibility level of typhoid fever in Ethiopia: A systematic review and meta-analysis

**DOI:** 10.1016/j.pmedr.2021.101670

**Published:** 2021-12-13

**Authors:** Melese Yeshambaw Teferi, Ziad El-Khatib, Endawoke Amsalu Alemayehu, Hawult Taye Adane, Azeb Tarekegn Andualem, Yonas Abebe Hailesilassie, Abraham Sahilemichael Kebede, Benedict Oppong Asamoah, Minyahil Tadesse Boltena, Mulatu Biru Shargie

**Affiliations:** a Armauer Hansen Research Institute, Ministry of Health, Ethiopia; b Department of Global Public Health, Karolinska Institutet, Stockholm, Sweden; c World Health Programme, Université du Québec en Abitibi-Témiscamingue (UQAT), Québec, Canada; d Social Medicine and Global Health, Department of Clinical Sciences, Lund University, Sweden; e University of Brighton, School of Sports and Health Sciences, United Kingdom

**Keywords:** AHRI, Armauer Hansen Research Institute, AMS, Antimicrobial Susceptibility, AMR, Antimicrobial Resistance, XDR, Extensive Drug Resistance, JBI, The Joanna Briggs Institute, LMICs, Low- and Middle-Income Countries, PRISMA, Preferred Reporting Items for Systematic Reviews and Meta-analyses, PROSPERO, International Prospective Registry of Systematic Reviews, SSA, Sub-Saharan Africa, WHO, The World Health Organization, Antimicrobial resistance, Antimicrobial susceptibility, Typhoid fever, Systematic review and Meta-analysis, Ethiopia

## Abstract

Typhoid fever continues to be a health challenge in low-and middle-income countries where access to clean water and sanitation infrastructure is scarce. The non-confirmatory diagnostic method continues to hinder effective diagnosis and treatment, ensuring in a high antimicrobial resistance. This systematic review and *meta*-analysis aimed to estimate the pooled prevalence and antimicrobial susceptibility level of typhoid fever in Ethiopia.

The review was designed based on the condition-context-population review approach. Fifteen eligible articles were identified from PubMed, Google Scholar, and Science Direct databases. Risk of bias and quality of studies were assessed using the Joanna Briggs Institute’s appraisal criteria. Heterogeneity was assessed using Cochran’s Q test and *I^2^* statistics. The review protocol was registered in PROSPERO (registration number CRD42021224478).

The estimated pooled prevalence of typhoid fever from blood and stool culture diagnosis was 3% (95% CI: 2%–4%, *p < 0.01*) (*I^2^* = 82.25) and Widal test examination 33% (95% CI: 22%–44%) (*I^2^* = 99.14). The sub-group analyses identified a lower detection of typhoid fever of 2% (95% CI: 1%–3%) among febrile patients compared to typhoid suspected cases of 6% (95% CI: 2%–9%). The stool culture test identified was twofold higher, value of 4% (95% CI: 2%-7%) *salmonella* S. Typhi infection than blood culture test of 2% (95% CI: 1%–4%). The antimicrobial susceptibility of *salmonella* S. Typhi for antibiotics was 94%, 80% and 65% for ceftriaxone, ciprofloxacin, and gentamycin respectively. Low susceptibility of *salmonella* S. Typhi isolates against nalidixic acid 22% (95% CI: 2%–46%) and chloramphenicol 11% (95% CI: 2%–20%) were observed. The diagnosis of typhoid fever was under or overestimated depending on the diagnostic modality. The Widal test which identified as nonreliable has long been used in Ethiopia for the diagnosis of *salmonella* S. Typhi causing high diagnosis uncertainties. Antimicrobial susceptibility of *salmonella* S. Typhi was low for most nationally recommended antibiotics. Ethiopian Food and Drug Authority must strengthen its continued monitoring and enhanced national antimicrobial surveillance system using the best available state-of-the-art technology and or tools to inform the rising resistance of *salmonella* S. Typhi towards the prescription of standard antibiotics. Finally, it is crucial to develop an evidence-based clinical decision-making support system for the diagnosis, empiric treatment and prevention of antimicrobial resistance.

## Highlights

• The diagnosis of typhoid fever was under or overestimated depending on the diagnostic modality.
• Widal test is none reliable diagnosis method of typhoid fever causing high diagnosis uncertainty.
• *Salmonella* S. Typhi was resistant for most nationally recommended antibiotics in Ethiopia.
• Continued monitoring and surveillance is needed to inform the rising resistance of S. Typhi.
• Evidence-based decision-making on the diagnosis and resistance of typhoid fever is crucial.

## 1 Introduction

Typhoid fever continues to be a health challenge and health security threat to low- and middle-income countries (LMIC) due to inadequate access to clean water and poor sanitation infrastructure (Akinyemi et al., 2018, Sur et al., 2018, Britto et al., 2018). The global prevalence of typhoid and paratyphoid fever was estimated to be over 14 million and the highest burden of the infection were reported from sub-Saharan Africa (SSA) (The global burden of typhoid and paratyphoid fevers, 2019, Marchello et al., 2019). According to a recent multi-centered population-based surveillance, *Salmonella* spp accounted for more than 33% of infections from all bacterial pathogens in SSA and S. Typhi were the most common 24% isolates among *salmonella* spp (Marks et al., 2017). Reports show the highest incidence of typhoid fever among children of age 2–4 years (Britto et al., 2018, Marks et al., 2017, Antillón et al., 2017).

A five-year retrospective study revealed that the prevalence of typhoid fever diagnosed by Widal test among patients of all age categories has increased fourfold, between years 2014 and 2018, ranging from 9.2 to 37.4% (Lemi et al., 2019). The prevalence of typhoid fever confirmed by the detection of its biomarker using blood culture among febrile patients were estimated to be 5%, which is significantly lower than the conventional Widal test result (Habte et al., 2018). Among a 288 collected blood samples, nearly half were positive in Widal test, and only 1 (0.7%) yielded *Salmonella* isolates during blood culture (Garedew et al., 2018). In addition, a study conducted among febrile patients in Southern Ethiopia estimated the prevalence of typhoid fever through the detection of the pathogen using blood culture to be 1.6% (Awol et al., 2021).

Poor diagnosis continues to hinder effective control of concurrent typhoid fever due to non-specific clinical presentation of the disease, lack of resources, insufficient access to health facilities, and lack of trained health care providers (Uneke, 2008). Symptoms related to febrile illness are often misdiagnosed in LMICs where proper diagnostic tools are not available (Zerfu et al., 2018). Assessment of the burden of typhoid fever in endemic areas are limited to rapid serological tests which has low degree of sensitivity and specificity making the confirmed typhoid fever cases unreliable (Ajibola et al., 2018). Countries with endemic incidence of typhoid fever lack well-established population-based national surveillance systems which created substantial knowledge gap to inform policy and impact the healthcare practice (Radhakrishnan et al., 2018).

Antimicrobial resistance (AMR) is an emerging public health concern due to inappropriate use of antimicrobial agents, self-medication, and lack of clinical diagnostic tools to support antibiotic de-escalation in LMIC (Bebell and Muiru, 2014, Castro-Vargas et al., 2020, Varma et al., 2018). AMR of *Salmonella* S. Typhi has initially emerged to the traditional first-line drugs such as chloramphenicol, ampicillin, and trimethoprim-sulfamethoxazole (Crump, 2019, Legese, et al., 2018, Amsalu et al., 2021). A study conducted in sub-Saharan Africa (SSA) identified high incidences of MDR S. Typhi in children aged < 15 years (Park et al., 2018) and similar report from Pakistan stated high multidrug-resistant (MDR) isolates,76% and significant Extensive drug resistance (XDR) *Salmonella* S. Typhi (Dyson et al., 2019, Hussain et al., 2019). Now XDR *Salmonella* S. Typhi is a major threat in Asia, while MDR has been expanding in SSA (Akram et al., 2020, Marchello et al., 2020).

Antibiotic prescription without confirmatory diagnostic modality directly contributes to disease severity and high AMR (Qamar et al., 2018). Infection with resistant microorganisms has severe health outcomes including longer illnesses, increased mortality and morbidity (9789241509763_eng.pdf). Clinically determining which patients require antibacterial drugs and susceptible to empirical antibacterial have been identified as a challenge for clinicians in Ethiopia (Garedew et al., 2018). The national typhoid fever surveillance system data is lacking on typhoid fever diagnosis uncertainties and associated morbidity that could inform policymakers for evidence-based decision making and impacting population practice for the prevention of typhoid fever and its antimicrobial susceptibility. Therefore, this systematic review and *meta*-analysis aimed to produce the proportion *salmonella* S. Typhi in different diagnostic modalities and associated antimicrobial susceptibility of typhoid fever in Ethiopia.

## 2 Methods

The overall review approach was designed based on the condition-context-population (CoCoPop) review method. Each section of the review was done and reported according to the Preferred Reporting Items for Systematic Reviews and Meta-Analyses (PRISMA) guideline (Moher et al., 2009). The review protocol has been registered in the International Prospective Register of Systematic Reviews (PROSPERO) under the registration number CRD42021224478.

### 2.1 Search strategies

The literature search was performed from during 1–30 February 2021. Studies published in the English language and conducted in Ethiopia from January 2010 through February 2021 were eligible for this review. Original studies providing information on the proportion of typhoid fever and antimicrobial susceptibility status were identified from PubMed, Google Scholar, and Science Direct databases. Terms within the same concepts were connected with Boolean “OR” and combined with other components of search terms using Boolean “AND”. The final search terms was built using a combination of keywords and search terms, “(((((((((Prevalence) OR Incidence) OR frequency) OR morbidity) OR burden) AND typhoid) OR typhoid fever) OR salmonella Typhi) OR S Typhi) AND Ethiopia”, to identify studies and citation searching from identified articles to avoid exclusion of relevant articles.

### 2.2 Study selection process

All identified articles from the different databases were imported to the Endnote reference manager. After the removal of duplicates three levels of screening based on title, abstract, and full-text review were performed. Articles that were not fulfilling the criteria were excluded at any level of the title, abstract or full-text review based on the eligibility criteria. A full-text review was conducted for articles eligible for the title and abstract review. A detailed full-text review was conducted to find out potential articles on prevalence and AMR of typhoid fever. The methodological quality assessments were conducted using the Joanna Briggs Institute (JBI) quality appraisal checklist, and studies judged to be of high quality were included in the analysis.

### 2.3 Eligibility criteria

Cross-sectional studies which reported the proportion of typhoid fever using the widal test or culture based diagnosis and AMR status in Ethiopia that are published in English language from January 2010 through February 2021 were included in the review. Exclusion criteria: Studies were excluded if the full article was inaccessible, conducted outside Ethiopia, systematic reviews, or randomized controlled trials.

### 2.4 Data extraction and review process

Full-length articles of the selected studies were screened against the inclusion criteria before the data extraction. Data extraction was performed by two authors (MYT and MTB) independently. The selected studies were reviewed to extract data such as year of publication, author(s), the geographical location of the study area, the period of study, study design, sample size, and proportion of typhoid fever and antimicrobial resistant status. Disagreement to include or exclude articles between the reviewers was resolved by the reviewer (HTA) to arrive at the final decision.

### 2.5 Methodological quality assessment

The two authors (MYT and MTB) independently assessed the methodological quality of included studies. The risk of bias and the overall quality of included studies was evaluated according to the JBI quality appraisal tool for prevalence and incidence studies (Kim et al., 2014) (see Supplementary Table A).

### 2.6 Statistical analysis and heterogeneity

Meta-analysis was carried out using metaprop command of STATA version 14 (Stata Corp LP, College Station, TX, USA) that used to estimate the proportion of *salmonella* S. Typhi using culture and Widal diagnosis. Heterogeneity between studies was evaluated using Cochran’s Q test and the *I^2^* statistic. Random-effects *meta*-analyses were used to combine the results of studies and were measured as proportions of typhoid fever and antimicrobial susceptibility level with 95% CI. Statistical analyses were carried out using STATA Version 14 software. The detailed descriptions of the original studies are presented in a table (Table 1).

**Table 1:** Characteristic of the included study for systematic review and meta-analysis.

id	authors	Location	Study Design	Type participants	Sample size	Male	Female	Widal test	Blood Cult	Stool cult	CRD	NA	CHL	CIP	GM
1	Awol, R.N., (Awol et al., 2019)	SNNP	CS	Febrile	381	172	209		6		6	0	5	5	
2	Teshome, B., et al (Teshome, 2019)	Oromia	CS	Diarrheal	232	99	133			9		4	4	7	
3	Admassu, D. (Admassu et al., 2019)	Jigjiga	CS	Febrile	200		.			14	9	5	0	10	9
4	Habte, L., et al., (Habte, 2018)	Oromia	CS	TF Suspected	421	186	235		21						
5	Garedew, L., et al., (Garedew et al., 2018)	Addis Ababa	CS	TF Suspected	367	220	147	148	1	8					
6	Zerfu, B., et al. (Zerfu et al., 2018)	Afar	CS	Febrile	630	253	397	83							
7	Ameya, G., et al (Ameya et al., 2017)	SNNP	CS	TF Suspected	95	46	49	65		19					
8	Wlekidan, L.N., et al., (Wlekidan et al., 2015)	Tigray	CS	Febrile	502	245	269	343	8		6			7	5
9	Feleke, S.M., A (Feleke et al., 2015)	Oromia	CS	Febrile	280	104	176	52							
10	Andualem, G., et al., (Andualem et al., 2014)	Addis Ababa	CS	TF Suspected	270	84	186	88	11						
11	Tadesse, H. (Tadesse and Tadesse, 2013)	Tigray	CS	Febrile	398	176	222	41							
12	Birhanie, M., et al. (Birhanie et al., 2014)	Amhara	CS	Febrile	200	120	80	38	1						
13	Deksissa, T. and E.Z (Deksissa and Gebremedhin, 2019)	Oromia	CS	Febrile	372	152	220	184		3	3		2	2	2
14	Weyesa, J.B. (Weyessa, 2014)	Addis Ababa	R CS	Febrile	4872	2793	2079	686							
15	Amsalu, T., C. Genet (Amsalu et al., 2021)	Amhara	CS	Febrile	150	69	81			6	5	1	0	5	4

Key: CRO = Ceftriaxone, GM = Gentamicin, CIP = Ciprofloxacin, CHL = Chloramphenicol, NA = Nalidixic acid, SNNP = southern Nation Nationalities Peoples.

## 3 Results

### 3.1 Search result

**Fig. 1:**
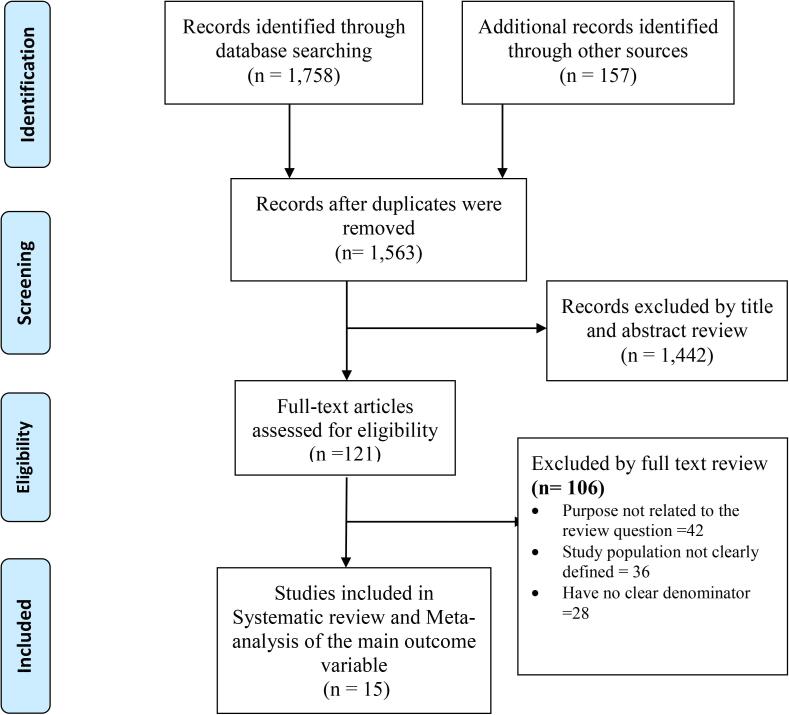
PRISMA flow diagram shows the search strategy and screening of eligible studies at different levels of the review process.

A total of 1758 articles were identified. A total of 1563 articles were non-duplicate and subjected to further evaluation. Then 1442/1563 (92.3%) articles were excluded based on the title and abstract screening, leaving 121 to be retained for detailed full-text review. After full-text evaluation, 15/121 (12.4%) articles were found to be eligible (Fig. 1)

### 3.2 Characteristics of included studies and study participants

The sample size of the studies ranged from 95 in Southern Nations Nationalities of People’s Region (SNNPR) (Ameya et al., 2017) to 4,872 in the capital city, Addis Ababa (Weyessa, 2014). From the total of 15 articles, only 6 (40%) reported the prevalence of typhoid fever and the AMR level (Habte et al., 2018, Garedew et al., 2018, Awol et al., 2021, Zerfu et al., 2018, Amsalu et al., 2021, Ameya et al., 2017, Weyessa, 2014, Amsalu et al., 2021, Teshome et al., 2019, Feleke et al., 2015, Tadesse and Tadesse, 2013, Wlekidan et al., 2015, Deksissa and Gebremedhin, 2019, Andualem et al., 2014, Birhanie et al., 2014, Admassu et al., 2019). A total of 9,370 study participants were included. Majority, (n = 7985/9,370; 85.22; (Awol et al., 2021, Zerfu et al., 2018, Amsalu et al., 2021, Weyessa, 2014, Feleke et al., 2015, Tadesse and Tadesse, 2013, Wlekidan et al., 2015, Deksissa and Gebremedhin, 2019, Birhanie et al., 2014, Admassu et al., 2019) participants were febrile patients and (1385/9,370, 14.78; (Habte et al., 2018, Garedew et al., 2018, Ameya et al., 2017, Andualem et al., 2014) were typhoid suspected cases. Most of the studies (n = 11/15; 73.3%) involved suspected typhoid patients who had some or all of the following symptoms (history of fever for ≥ 2 days, body temperature greater than 37.5 °C, abdominal pain, headache, constipation or diarrhea, fatigue, headache, joint, and back pain) (Habte et al., 2018, Awol et al., 2021, Zerfu et al., 2018, Amsalu et al., 2021, Ameya et al., 2017, Feleke et al., 2015, Tadesse and Tadesse, 2013, Wlekidan et al., 2015, Deksissa and Gebremedhin, 2019, Andualem et al., 2014, Birhanie et al., 2014). Some of the studies (4/15) also considered taking antibiotic treatment for the last two weeks in average as inclusion) and exclusion criteria (Ameya et al., 2017, Wlekidan et al., 2015, Deksissa and Gebremedhin, 2019, Birhanie et al., 2014).

Eleven studies used culture technique for the diagnosis of typhoid fever: 6 used stool culture (Garedew et al., 2018, Amsalu et al., 2021, Ameya et al., 2017, Teshome et al., 2019, Deksissa and Gebremedhin, 2019, Admassu et al., 2019) and 5 used blood culture to identify *salmonella* S. Typhi (Habte et al., 2018, Garedew et al., 2018, Awol et al., 2021, Wlekidan et al., 2015, Andualem et al., 2014, Birhanie et al., 2014). Among the included studies, 10 of them used Widal test as a diagnostic method (Garedew et al., 2018, Zerfu et al., 2018, Amsalu et al., 2021, Ameya et al., 2017, Weyessa, 2014, Feleke et al., 2015, Wlekidan et al., 2015, Deksissa and Gebremedhin, 2019, Andualem et al., 2014, Birhanie et al., 2014). While 3 studies used the Widal tube agglutination test (titration) to diagnose typhoid fever (Garedew et al., 2018, Zerfu et al., 2018, Wlekidan et al., 2015) and five studies used the slide agglutination test method (Zerfu et al., 2018, Ameya et al., 2017, Weyessa, 2014, Deksissa and Gebremedhin, 2019, Birhanie et al., 2014). Only two studies employed combined slide agglutination and tube agglutination tests at the same time (Feleke et al., 2015, Andualem et al., 2014). Six studies used Widal and culture-based diagnosis at the same time and see the variation of the result (Garedew et al., 2018, Ameya et al., 2017, Wlekidan et al., 2015, Deksissa and Gebremedhin, 2019, Andualem et al., 2014, Birhanie et al., 2014) (Table 1).

### 3.3 Meta-analysis

**Fig. 2:**
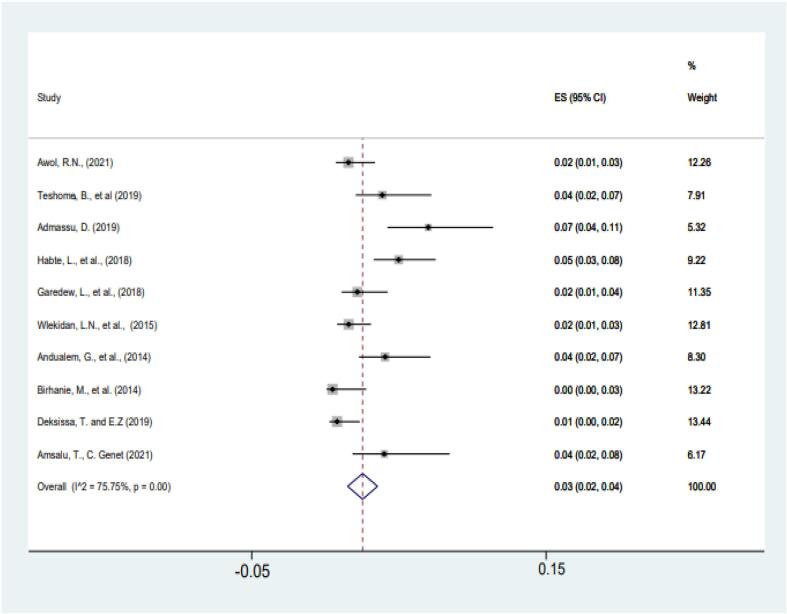
Culture-based estimated pooled prevalence of typhoid fever, from 2010 to 2021 in Ethiopia.

The pooled prevalence of typhoid fever based on the eligible studies were 3% (95% CI: 2%–4%, *p < 0.01*) (reported blood and stool culture diagnosis result) (Fig. 2). The heterogeneity test indicated that all studies on typhoid prevalence had significant heterogeneity (*I^2^* = 82.25). Therefore, the random-effects model was used for the *meta*-analysis.

### 3.4 Sub-group analysis

**Fig. 3:**
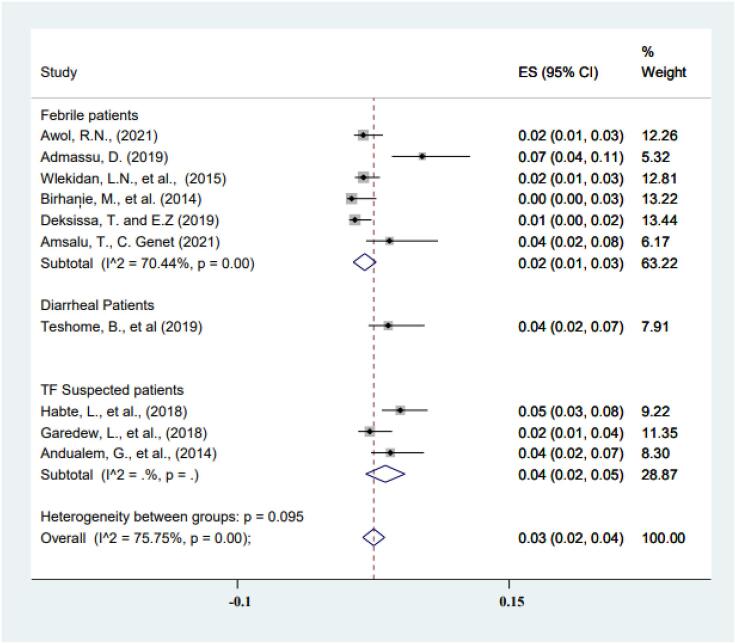
Subgroup analysis of typhoid fever prevalence based on the type of the study participants.

**Fig. 4:**
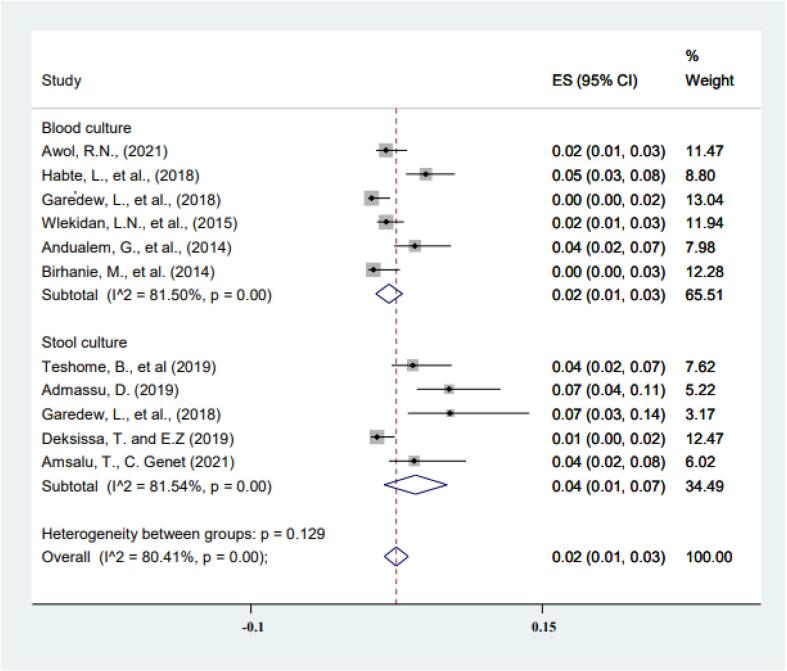
Subgroup analysis of typhoid fever prevalence based on the type of culture diagnosis.

Fig. 3 shows the sub-group analyses of prevalence of typhoid fever based on the types of the study participants. Accordingly, the prevalence of typhoid fever was 2% (95% CI: 1%–3%) in febrile patients compared to 6% (95% CI: 2%–9%) of typhoid suspected patients with *I^2^* values of 70.44% and 85.92%, Fig. 3**.** The subgroup analysis based on the culture technique used for diagnosis of typhoid fever showed two times higher proportion of *salmonella* S. Typhi identified based on stool culture test 4% (95% CI: 2%–7%) compared to 2% (95% CI: 1%-4%) on blood culture test Fig. 4.

**Fig. 5:**
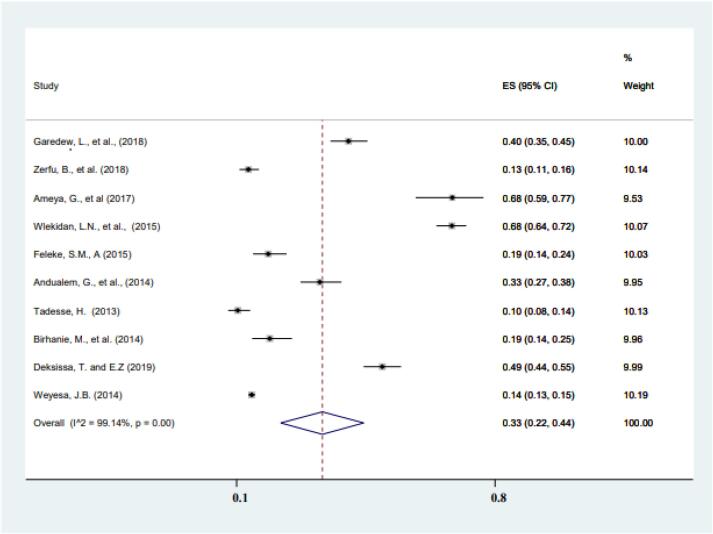
Widal test based estimation of typhoid fever, from 2010 to 2021 in Ethiopia.

Fig. 5 shows the pooled prevalence of typhoid fever using Widal test. The prevalence of typhoid fever based on the Widal test diagnostic modality was 33% (95% CI: 22%–44%), which is higher compared to the prevalence of typhoid fever identified based on the blood and stool culture. The random-effect model was used for the *meta*-analysis as a response to the significant heterogeneity between the studies (*I^2^* = 99.14).

### 3.5 Publication bias

**Fig. 6:**
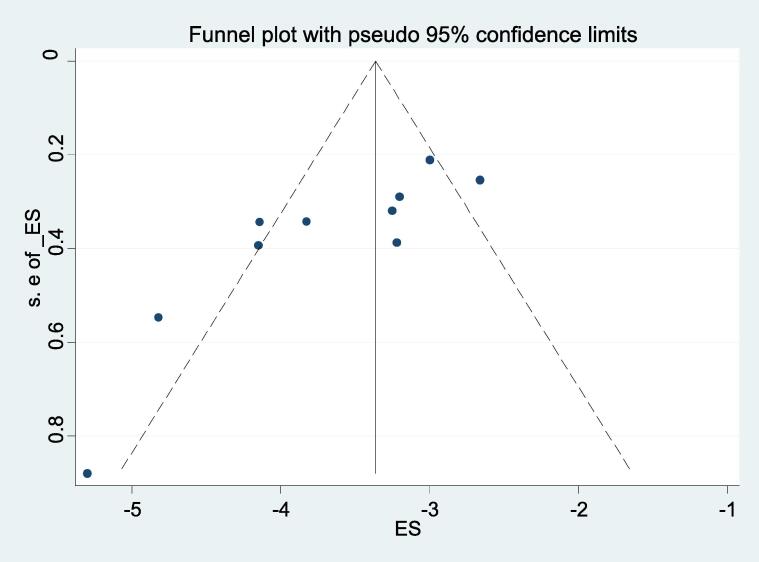
Funnel plots of standard error or precision used to assess publication bias.

Publication bias was assessed using funnel and egger's test. The funnel plot was constructed from study estimates with a pseudo 95% confidence limit against the standard error of the estimates, revealed minimal publication bias (Fig. 6). The Egger’s test indicated that there is a high publication bias (p < 0.01) on studies reporting the prevalence of *salmonella* S. Typh in human blood and stools in Ethiopia. The in agreement between the funnel plot and the egger’s test for the possible publication bias might have arised from the limitation of eggers test to detect bias when the numbers of studies are small.

### 3.6 Antimicrobial susceptibility level

Antimicrobial susceptibility data of typhoid fever were extracted from six included articles. The level of antimicrobial susceptibility of *salmonella* S. Typhi isolates were analyzed for five commonly prescribed antibiotics. The *meta*-analyses revealed that a high-pooled susceptibility level of *salmonella* S. Typhi with 94% (95% CI: 85%–102%) ceftriaxone, 80% (95% CI: 68%–91%) for ciprofloxacin, and 65% (95% CI: 48%–81%) of gentamycin. However, a low pooled susceptibility level of *salmonella* S. Typhi isolates was identified against nalidixic acid to 22% (95% CI: 2%–46%) and chloramphenicol 11% (95% CI: 2%–20%) (Table 2).

**Table 2:** Show the antimicrobial susceptibility level of the commonly reported antimicrobials in Ethiopia.

Antibiotics	N of studies	I^2^	ES (95% CI)
Ceftriaxone	5	64.14%	0.94 (0.85, 1.02)
Ciprofloxacin	6	0.00%	0.80 (0.68, 0.91)
Gentamycin	4	0.00%	0.65 (0.48, 0.81)
Nalidixic acid	4	80.63%	0.22 (0.02, 0.46)
Chloramphenicol	5	89.60%	0.11 (0.02, 0.20)

I^2^ = I square statistics ES = Estimates; CI = Confidence Interval.

## 4 Discussion

This review estimated the pooled prevalence of typhoid fever over the period of ten year published (2010 to 2021) in Ethiopia. Eleven studies which reported the magnitude of typhoid fever confirmed by blood culture and stool specimen, and four additional studies that specifically used Widal test were candidates for *meta*-analysis. The pooled prevalence of typhoid fever in this study was 3. The finding of this study was slightly higher than the pooled estimate of *salmonella* S.Typhi isolates among of febrile participant in five Asian countries (2%; n = 23750 study participants) (Ochiai et al., 2008). The finding of this review was in agreement with a study in Cameroon that reported 2.5% culture confirmed *salmonella* S. Typhi among febrile patients (Nsutebu et al., 2003).

However, the finding from the current study was lower than a report from Nepal (4.1%) (Andrews et al., 2018), and India (4.4%) (Bhattacharya et al., 2011), Egypt (5%) (Srikantiah et al., 2006) of culture-confirmed typhoidal *Salmonella* infection among those with a provisional clinical diagnosis. A recent study among typhoid suspected Nigerian patients reported 5.2% of culture confirmed *salmonella* S. Typhi which is higher compared to this finding (Ohanu, 2019). The finding from the current study is lower than studies reported higher proportion of *Salmonella* S. Typhi isolates in South Asia (8.8%) (Zellweger et al., 2017), India (9.7%) (Mengist and Tilahun, 2017), Nepal (9.2%) (Ohanu, 2019), Nigeria (14.1%) (Mawazo et al., 2019), and Vietnam (14.5%) (Tran et al., 2005). The reason for the difference in the presence of the *salmonella* S. Typhi may include but not limited to the poor health care utilization, lack of trained health professions, inadequate diagnostic modality, low socio-economic status, and unavailability of clean water.

The subgroup analyses based on the type of study participants involved in this study showed a considerable difference in the prevalence of typhoid fever. Accordingly, the proportion of typhoid fever among typhoid suspected patients was 6% (95% CI: 2 –9%), which is three times higher than febrile patients 2% (95% CI: 1%–3%). The finding of the current study is supported by a report in Ethiopia identified low contribution of *Salmonella* S. Typhi, 1.6% for febrile patients with 21.3% other non-*Salmonella* pathogenic bacteria isolates (Wlekidan et al., 2015, Teferi et al., 2019). High proportion of the *Salmonella* S. Typhi among typhoid suspected patients compared to the febrile patients may shows the fact that suspected patients have high probability of *salmonella* S. Typhi positive finding than febrile patients.

The subgroup analysis based on the type of culture test for the diagnoses of typhoid fever revealed that the rate of *Salmonella* Typhi identified based on stool culture test was two times higher than the isolates identified by the blood culture test 4% and 2% respectively. The finding of high level of *salmonella* S. Typhi isolates based on the stool culture diagnosis compared to the blood culture may be an indicator of high chronic carriers’ status of the participants (Abate and Assefa, 2021). However, our finding showed lower stool culture positivity of *salmonella* S. Typhi against the 7.6% from a study conducted among outpatients in Ethiopia (Abate and Assefa, 2021) and 11% of typhoid suspected patients from Tanzania (Mawazo et al., 2019).

The proportion of typhoid fever based on the Widal test diagnosis was 33% which is higher than the pooled prevalence identified based on the blood and stool culture diagnosis, 3%. The finding of the current review indicated higher *salmonella* S. Typhi than a report from Nigeria, 24.5% (OR et al., 2015) and lower than a result from Tanzania (81%) (Mawazo et al., 2019). The main reason for this considerable difference between Widal and culture tests may be due to poor reliability of the Widal test to indicate the true test value (Mengist and Tilahun, 2017). A comparative study on Typhoid diagnosis revealed that the Widal test has a low specificity with sensitivity (84.2%), specificity (35.5%), PPV (24.6%) and NPV (90.0%) of slide agglutination test against stool culture were (Ameya et al., 2017). Similarly other studies concluded that Widal test is not reliable for diagnosis of typhoid fever since false positive and negative results are common (Ohanu, 2019, Mengist and Tilahun, 2017, Mawazo et al., 2019)

This disagreement of typhoid fever detection using Widal test versus culture based diagnosis could be attributed to the poor diagnostic tools resulted and sparse local disease data which are not well-integrated as a locally generated evidence for clinical decision making (Steele et al., 2016). Ethiopia has been using Widal test as a diagnostic modality for typhoid fever detection and this contributes to the emerging antimicrobial resistance due to misdiagnosis and the associated drug prescription (Wlekidan et al., 2015, Animut et al., 2009).

### 4.1 Antimicrobial susceptibility level

This review described *salmonella* S. Typhi isolates had different susceptibility profiles against selected antimicrobial agents. The *meta*-analyses revealed that the pooled susceptibility level of *salmonella* S. Typhi was 94% for ceftriaxone, 80% for ciprofloxacin, and 65% for gentamycin. The finding of this study showed similar susceptibility of *salmonella* S.Typhi to ceftriaxone in Tanzania 95.6% (Ohanu, 2019). In line with this, a review reported higher *salmonella* S. Typhi susceptible to ceftriaxone (Crump, 2019). Our findings have a slight disagreement with hospital-based study conducted in India, which reported 100 % susceptibility of *salmonella* to ceftriaxone (Sharma et al., 2018, Bernabe et al., 2017). The susceptibility level 80% of ciprofloxacin identified by this review is low compared to 100% susceptibility level reported from Tanzania (Marchello et al., 2020, Ohanu, 2019, Sharma et al., 2018) and 98.5% in Kenya (Breiman et al., 2012). However, the finding of the recent report from India indicated a lower susceptibility level of *salmonella* for ciprofloxacin 71.3% (Sharma et al., 2018).

The finding of this study revealed that the susceptibility of *salmonella* S. Typhi for gentamycin were 65%, which is higher than the worldwide AMR 11.0% (Marchello et al., 2020). According to the finding of this systematic review, the susceptibility of *salmonella* S. Typhi for nalidixic acid was 22%, which is higher than a study conducted in Vietnam with 19.6%, and lower than a report from 81.6% Bnagladesh (Chiou et al., 2014), and 93.2% Kenya (Breiman et al., 2012). The result of this review identified lower susceptibility of *salmonella* S. Typhi for chloramphenicol 11%, which is in agreement with 17.4% in Kenya (Breiman et al., 2012). Also, this finding was supported by the 80.4% resistance in Vietnam (Chiou et al., 2014).

However, the finding of this systematic review was lower than reports of susceptibility of *salmonella* S. Typhi for chloramphenicol worldwide (25.9%) (Marchello et al., 2020), West Africa (38.3%) (Bernabe et al., 2017), Egypt (33%) (Srikantiah et al., 2006) India (87.9%) (Sharma et al., 2018), and 66.8% in Pakistan (Qamar et al., 2014). The difference in susceptibility of *salmonella* S. Typhi to chloramphenicol may be due to wrong drug prescription without confirmatory diagnosis, lack of proper diagnostic tools, and insufficient access to trained health care providers and facilities (Zerfu et al., 2018, Radhakrishnan, et al., 2018, Brink et al., 2016).

This review reported widespread multidrug resistant *salmonella* S. Typhi, i.e. resistance to more than two antimicrobials, which the pathogen developed MDR up to 66.7% of isolates (Amsalu et al., 2021, Deksissa and Gebremedhin, 2019, Admassu et al., 2019). These findings were in agreement with the review report of MDR in SSA (32.6%) (Wang et al., 2021) and a population-based study in a rural Kenya which indicated a (75%) multi-drug resistant S. Typhi isolates (Breiman et al., 2012).

The overlapping clinical features of viral and bacterial infections dramatically reduce the ability of clinicians to distinguish which patients would benefit from an antibiotic or not (Ethiopia_-_General_Hospital_CPG.PDF). A study conducted in Ethiopia stated that patients received inappropriate treatment due to wrong diagnosis based on empirical symptoms, clinical signs, and tube Widal test (Garedew et al., 2018). A report also conclude that physicians perceived a higher frequency of diagnostic uncertainty resulting in higher antibiotic use (Wasihun et al., 2015). As a result of this recommended drugs according to the Ethiopian national standard treatment guideline; chloramphenicol, ciprofloxacin, gentamicin including ceftriaxone were identified in different level of resistant to typhoid fever [68]. This emerging drug resistance of all of the recommended antibiotics may be due to the indiscriminate drug prescription [69]. This urges highly intensified effort of national and global level policy makers to develop interventional strategy that improves the quality of the diagnosis as miss-diagnosis fuels antimicrobial resistance and drug side effect.

### 4.2 Study limitations

Despite such crucial findings, this study had limitations, studies included for analysis were involve participants with different clinical presentations, age groups, and background status; and the high degree of heterogeneity among the studies was also another limitation of the review.

## 5 Conclusion and recommendations

The systematic review results show that diagnosis of typhoid fever using the Widal test is prone to error with overestimated 33% high *salmonella* S.Typhi compared to a low 3% culture-based pooled prevalence in Ethiopia. This uncertainty in the diagnosis of *salmonella* S. Typhi leads to unnecessary antimicrobial prescription and subsequent antimicrobial resistance. The review also identified a low AMR of *salmonella* S. Typhi for nationally recommended drugs in Ethiopia. The Widal test which has long been used in Ethiopia for the diagnosis of *salmonella* S. Typhi is not reliable and confirmatory diagnosis modality that supports clinicians to identify the cause of an acute febrile illness. We suggest continued monitoring and enhanced national antimicrobial surveillance system using the best available state-of-the-art technology and or tools to inform the rising resistance of *salmonella* S. Typhi towards the prescription of standard antibiotics using essential drug list and develop evidence-based clinical decision-making support system for the empiric treatment and prevention of antimicrobial resistance. Emphasis should be given on developing a rapid, confirmatory, feasible and affordable diagnostic tool which is capable of detecting *Salmonella* S. Typhi infection and differentiating it from other infections.

## 6 Ethics approval and consent to participate

Not applicable.

## 7 Consent for publication

Not applicable.

## 8 Availability of data and material

The datasets during and/or analyzed during the current study are available from the corresponding author on reasonable request.

## 9 Fundingss

Not applicable.

## 10 Authors’ contributions

MYT was involved in a principal role in the conception of ideas, developing methodologies, analysis and writing the article. ZEK, EAA, HTA, ATA, YAH, BOA, MTB, MBS were participated in data interpretation, writing and revising. All authors read and approved the final version of the manuscript.

## Declaration of Competing Interest

The authors declare that they have no known competing financial interests or personal relationships that could have appeared to influence the work reported in this paper.
