# Supplementary material for: Prevalence and antimicrobial susceptibility level of typhoid fever in Ethiopia: A systematic review and meta-analysis
Source: Prev Med Rep. 2021 Dec 13;25:101670. doi: 10.1016/j.pmedr.2021.101670 (PMC8686025; doi:10.1016/j.pmedr.2021.101670)
Supplement: Supplementary data 1 [file mmc1.docx]

Supplementary Table (Supporting information); Shows study level risk of bias measurement score using the Joanna Briggs Institute (JBI) critical appraisal assessment tool

| **Included studies** | **Reviewer #1** | **Total No Yes (Y)** | **Reviewer #2** | **Total No Yes (Y)** | **Average Yes (Y)** | **Percentage of Yes (Y)** | **Judgment** |
| --- | --- | --- | --- | --- | --- | --- | --- |
| Awol, R.N., 2021 | Y | 7 | Y | 7 | 7.0 | 77.80% | Low |
| Amsalu, T., C. Genet, 2021 | Y | 7 | Y | 8 | 7.5 | 83.30% | Low |
| Teshome, B., et al, 2019 | Y | 6 | Y | 6 | 6.0 | 66.70% | Moderate |
| Admassu, D., 2019 | Y | 8 | Y | 7 | 7.5 | 83.30% | Low |
| Deksissa, T. and E.Z 2019 | Y | 8 | Y | 7 | 7.5 | 83.30% | Low |
| Habte, L., et al., 2018 | Y | 7 | Y | 8 | 7.5 | 83.30% | Low |
| Zerfu, B., et al., 2018 | Y | 6 | Y | 6 | 6.0 | 66.70% | Moderate |
| Ameya, G., et al, 2017 | Y | 7 | Y | 6 | 6.5 | 72.20% | Low |
| Wlekidan, L.N., et al., 2015 | Y | 8 | Y | 8 | 8.0 | 88.90% | Low |
| Feleke, S.M., A, 2015 | Y | 7 | Y | 7 | 7.0 | 77.80% | Low |
| Andualem, G., et al., 2014 | Y | 7 | Y | 7 | 7.0 | 77.80% | Low |
| Garedew, L., N., 2018 | Y | 7 | Y | 7 | 7.0 | 77.80% | Low |
| Birhanie, M., et al., 2014 | Y | 8 | Y | 7 | 7.5 | 83.30% | Low |
| Weyesa, J.B. 2014 | Y | 7 | Y | 6 | 6.5 | 72.20% | Low |
| Tadesse, H. , 2013 | Y | 6 | Y | 6 | 6.0 | 66.70% | Moderate |
| **Subtotal** |  |  |  |  |  |  |  |
| **Y=Yes** |  |  | **77.41%** |  |  |  |  |
| **N = No** |  |  | **22.59%** |  |  |  |  |

***Note.*** Study level risk of bias is calculated from the domain of nine criteria
